# Recent advances in lineage differentiation from stem cells: hurdles and opportunities?

**DOI:** 10.12688/f1000research.12596.1

**Published:** 2018-02-23

**Authors:** Joke Terryn, Tine Tricot, Madhavsai Gajjar, Catherine Verfaillie

**Affiliations:** 1 Department of Development and Regeneration, Stem Cell Institute Leuven, KU Leuven, Belgium

**Keywords:** Stem cells, disease modeling, regenerative medicine, genome editing

## Abstract

Pluripotent stem cells have the property of long-term self-renewal and the potential to give rise to descendants of the three germ layers and hence all mature cells in the human body. Therefore, they hold the promise of offering insight not only into human development but also for human disease modeling and regenerative medicine. However, the generation of mature differentiated cells that closely resemble their *in vivo* counterparts remains challenging. Recent advances in single-cell transcriptomics and computational modeling of gene regulatory networks are revealing a better understanding of lineage commitment and are driving modern genome editing approaches. Additional modification of the chemical microenvironment, as well as the use of bioengineering tools to recreate the cellular, extracellular matrix, and physical characteristics of the niche wherein progenitors and mature cells reside, is now being used to further improve the maturation and functionality of stem cell progeny.

## Introduction

Stem cells have the remarkable property of long-term self-renewal, and at the same time they can give rise to progressively more lineage-committed cells. Pluripotent stem cells (PSCs) are multipotent as they can generate all mature cells of the body. Although murine PSCs were already isolated in the 1980s ^1^, it was not until 1998 that human PSCs (hPSCs) were first isolated from human blastocysts, termed embryonic stem cells (ESCs) ^2^. This accomplishment, and, even more so, the creation of so-called ‘induced pluripotent stem cells’, or iPSCs, from mouse ^3^ and then human ^4^ fibroblasts in 2006–2007, opened up the possibility of generating any cell type for regenerative medicine. In addition, the availability of human ESCs (hESCs) and iPSCs (collectively termed PSCs) now provides us with tools to better understand human development as well as create models to study human diseases. Fully exploiting that potential, however, remains challenging. Although new stem cell differentiation protocols are published on a weekly basis, many hurdles remain regarding how to create mature stem cell progeny. In this review, we will discuss the current state of the art of stem cell culture and lineage differentiation from stem cells. Current limitations in creating mature PSC progeny are steering investigators to explore novel avenues in stem cell research. We discuss the roads that are being taken and need to be taken to improve the functionality of PSC-derived human tissue cells and ultimately exploit the full potential of hPSCs.

## Pluripotency matters

The derivation and culture of hESCs and human iPSCs (hiPSCs) use conditions that differ from those used to isolate their murine counterparts. This results in the capture of cells at different stages of embryonic development. Murine PSCs can be isolated by using several culture methods from mouse embryos. These include culture on mouse embryonic fibroblasts or using mTeSR and LIF, resulting in a population of cells containing chiefly ESCs but also extra-embryonic endoderm progenitors ^5^. More recently, it has been demonstrated that murine ESCs can be isolated and maintained in a ‘naïve’ ground state (termed 2i/LIF conditions) that resembles pre-implantation pluripotency wherein fate allocation in specific cell populations has not yet occurred. Unlike their murine counterparts, human PSCs in standard culture conditions resemble post-implantation, ‘primed’ epiblast stem cells, wherein initial fate allocation has occurred ^6^. When post-implantation murine or rat embryos are used, a similar epiblast-like cell type can be isolated as well ^7^. Such primed human and mouse ESCs have already undergone X-chromosome inactivation. Moreover, the generation of hiPSCs does not lead to reactivation of the X-chromosome. This X-chromosome inactivation state, however, is unstable, as long-term culture of female ‘primed’ hESCs or hiPSCs has been shown to cause ‘X-chromosome erosion’; that is, the inactivation of the silenced X-chromosome is progressively lost. X-chromosome erosion is associated with decreased differentiation potential, and this is inherited by the differentiated progeny ^8– 11^. In addition, excessive X-chromosome skewing is frequently seen ^12– 15^, yielding skewed progeny that could manifest or lose disease phenotypes. For example, in the case of Duchenne muscular dystrophy, female carriers of a dystrophin gene mutation rarely manifest with muscular dystrophy because the random nature of X-chromosome inactivation leads to a mixture of cells expressing either the wild-type or the disease gene. In this case, *in vitro* X-chromosome skewing could misrepresent the *in vivo* situation ^16^. Together with the fact that primed cells are not lineage-neutral, this may be a major contributing factor to the variability observed within and between different cell lines ^17^.

**Figure 1.**
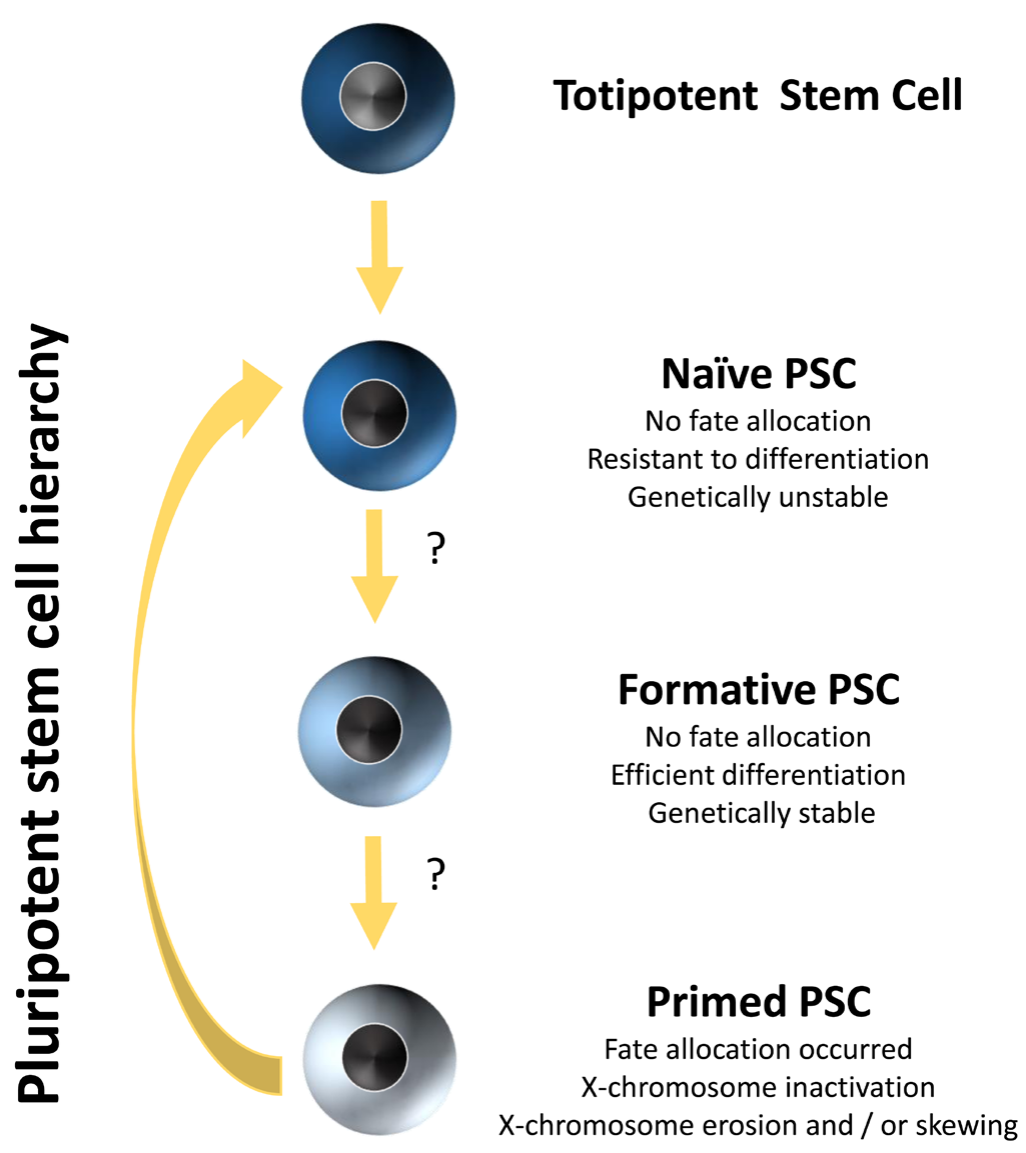
Pluripotent stem cell (PSC) hierarchy. Hypothesized hierarchy of human PSCs and their properties.

During the last 3 years, a number of protocols have been described in which primed hPSCs can be reset to a naïve state or which allow reprogramming of somatic cells to naïve hPSCs (reviewed in 18). However, such human naïve PSCs are more resistant to differentiation, and a step wherein naïve PSCs are committed to an intermediate primed state is required, at least in some studies ^19– 21^. For instance, transitioning through a more naïve state proved to be especially beneficial in the case of germ line cell differentiation. The initial fate allocation of regular primed hPSCs significantly dampens germ line competence, but converting hESCs to a more naïve state, using a protocol developed by Gafni *et al*. ^22^, drastically improved the efficiency of generating primordial germ cells ^23^. However, recent reports sound a cautionary note by reporting that naïve hPSCs are genetically unstable ^11, 24^. Hence, they are not good candidates to start lineage differentiation for either disease modeling or regenerative medicine. However, recent insights into the hierarchy of pluripotency during development have led to the identification of an additional cell population, to which the term ‘formative PSCs’ was assigned, that appears to be located in between naïve and primed PSCs ^25^ ( Figure 1). Such putative formative PSCs ^26^ are, like naïve PSCs, lineage-neutral; however, unlike naïve PSCs, they are hypothesized to be able to differentiate efficiently into all different cell lineages. Furthermore, they also appear to be genetically more stable than naïve hPSCs and, because they have higher levels of methyltransferase activities than primed PSCs ^27^, also may not be subject to X-chromosome erosion. Therefore, developing methods that allow capturing and maintaining hPSCs in this formative state will likely be of great importance to enable robust and controlled lineage differentiation.

## Lineage differentiation: current state of the art and shortcomings

To induce lineage differentiation from PSCs, investigators have used insights gained from development. Initially, this comprised the generation of embryoid bodies, wherein differentiation occurs spontaneously as a result of signals emanating from the different cell populations that spontaneously develop ^25^. To control the differentiation process better, subsequent studies have used step-wise addition of growth factors and cytokines, or inhibitors thereof, known to play a role during certain steps of differentiation, combined in the majority in cases with monolayer culture systems. This has enabled the generation of a number of cell types, including cells with features of neural subpopulations (for example, glutamatergic cortical neurons ^28^, cholinergic neurons ^29^, dopaminergic neurons ^30^, oligodendrocytes ^31^, and astrocytes ^32^), cardiac muscle ^33– 36^, or hepatocytes ^37– 39^, among many others. However, these cells resemble fetal tissue more than adult tissue in the majority of cases. Reasons for this are numerous and will be discussed below.

First, although we have some insight into the progressive maturation of cells in a tissue, this knowledge is based on quantitative reverse transcription polymerase chain reaction or genome-wide transcriptome studies throughout development, assessing gene expression in bulk populations of cells. However, as was already suggested by immunofluorescence staining for a limited number of marker proteins, not all cells in a tissue are equal. This is likely also true for developing cell populations, although such studies in human embryos are, for obvious reasons, not readily feasible. Nevertheless, thanks to the advent of single-cell RNAseq, single-cell variability in mature and developing tissues (the latter chiefly in mouse) can now be further elucidated (reviewed in 40, 41). For instance, it is now clear that neurons and astrocytes are brain region-specific, and transcriptional signatures for these are becoming available (reviewed in 42). Therefore, as has been done for neuronal differentiation protocols, different approaches will likely be needed to generate regionally specific astrocytes.

Second, although the mature phenotype of certain cells, such as hepatocytes, has been well established and can be used to define the maturation state of these cells, this is not the case for all cell types ^43^. Using astrocytes as an example, the definition of a ‘mature’ astrocyte remains unclear (reviewed in 44). Although in general the presence of glial fibrillary acidic protein or S100β or both has been used to define astrocytes, this is insufficient, as there are other neural precursors, such as radial glia, that could, on the basis of these markers alone, be classified as astrocytes ^45^. Therefore, markers or assays that reveal the functional properties of these cells, including propagation of calcium waves, glutamate handling, and inflammatory responses, should be used to define astrocytes ^46– 48^.

Third, standard culture conditions constitute a major roadblock in lineage differentiation, as they do not provide all of the necessary environmental cues. During embryogenesis, cells from different germ layers co-develop in response not only to graded and continuously changing concentrations of chemical factors generated by neighboring cells and cells at some distance ^49^ but also to cell–cell- and cell–extracellular matrix (ECM)-mediated signals. Although growth factor addition, in what we would call a ‘purist’ approach to a single-cell-type-at-a-time differentiation, tries to recreate the *in vivo* chemical signals, it is very crude and fails to recreate the subtle progressively changing levels of growth factors and morphogens present *in vivo*. Obviously, by attempting to make pure populations of differentiated cells, we preclude the influence on cell commitment and maturation of neighboring cells derived from the same or even a distinct germlayer. In addition, differentiation performed in 2D culture is generally in culture plastic wells coated with an ECM such as collagen, laminin, or matrigel. This does not start to recreate the complex and changing ECM or the physical characteristics of developing ‘soft’ organs. In addition, numerous other environmental cues affect cell differentiation—such as electrophysiological activity that regulates cardiac muscle or neural cell maturation ^50^; mechanical stimulation inducing cardiac differentiation ^51^; flow/shear force that affects endothelial differentiation ^52^; or nutrient composition of the blood that affects zonation of cells in the liver ^53^—all of which are only now starting to be evaluated to enhance lineage maturation.

Finally, the duration of many differentiation protocols does not reflect human gestation. It may not be surprising that differentiated progeny resembles fetal tissue more than adult tissue. For instance, during development, neurogenesis switches to gliogenesis late during gestation ^54^, which underlies the fact that very lengthy cultures are required to derive astrocytes from PSCs.

Another example is that PSC-derived hepatocytes usually harvested after 3–5 weeks continue to express the fetal hepatocyte gene, alpha-fetoprotein ^55^, and do not express markers associated with mature hepatic function, such as the detoxifying enzymes of the cytochrome P450 family (CYPs) ^56^. Although the fetal phenotype of PSC-derived hepatocytes allows disease modeling of, for instance, hepatotropic viruses such as dengue, hepatitis B, and hepatitis C ^57– 60^, their fetal metabolic signature limits their usefulness in drug toxicity studies ^43^. In contrast, in the field of regenerative medicine, the immaturity and plasticity of progenitor cells can be advantageous as, for example, dopaminergic neuronal progenitors still hold the ability to migrate and integrate when transplanted in Parkinson’s disease ^61^. Several clinical trials, conducted between 1990 and 2003, have tested the feasibility and clinical benefit of transplanting fetal mesencephalic grafts in the basal ganglia of patients with Parkinson’s disease ^61, 62^. Though not all equally successful, these trials served as a proof of principle and paved the way for trials using hPSC-derived progeny ^63^. Nevertheless, the use of incomplete differentiated progeny from PSCs may hold the risk of causing unwanted excessive proliferation ^64^ or even teratoma formation ^65^.

## Lineage differentiation: ways forward

### Genome editing

As stated above, the incomplete lineage differentiation from PSCs for many cell types is caused by our incomplete understanding of developmental processes. However, the newly available high-throughput single-cell transcriptomics and computational modeling of gene regulatory networks now allow in-depth characterization of cellular identity and lineage commitment, not only in adult tissues but also throughout development (the latter at least in mouse development) ^66– 68^. These studies start to identify previously underestimated heterogeneity in cell populations, in the adult as well as during development ^69, 70^, and in the meantime provide key targets and knowledge of transcriptional dynamics ^68^.

Such single-cell RNAseq data combined with novel genome engineering approaches, especially the Clustered Regularly Interspaced Short Palindromic Repeats (CRISPR) technology (reviewed in 71), allow investigators to ‘engineer’ PSC progeny toward specific lineages. CRISPR technology significantly enhances the frequency of homologous recombination in hPSCs. This is all the more important to create reporter cell lines, enabling the enrichment of precursors or mature cells from mixed populations. However, even more important for lineage differentiation approaches is the ability to exploit the CRISPR system to activate or interfere with gene expression, using CRIPSR-activation (CRISPRa) and CRIPSR-interfering (CRISPRi) approaches. For CRISPRa, a dead Cas (dCas), unable to cause double-strand breaks, is linked to a transactivator sequence, such as multiple herpes simplex virus protein VP16 repeats (VP64) ^72^, or VP64 fused with p65, a subunit of the ubiquitous NF-κB transcription factor complex (p65) and the Epstein-Barr virus R *transactivator (*Rta) (so-called VPR) ^73^, among others. The VPR transactivator, for instance, allows the activation of endogenous gene transcription by a median of 150-fold. Alternatively, the dCas can be fused with a gene repressor such as the Krüppel-associated box (KRAB) domain of *Kox1* ^74^ or four copies of the *mSin3* domain (termed SID4X) ^71^ to inhibit gene transcription by up to 15-fold. The CRISPR–dCas-activator/repressor can be integrated into a safe harbor locus ^75^ and be driven by different inducible promoters to enable the expression of either the transactivator or the repressor. In addition, the use of diverse dCas9 orthologues from different bacteria and their respective single-guide RNA ^76^ further enhances the possibilities for sequential or combinatorial induction (or both) of transcription factors (TFs) that are insufficiently expressed in PSC progeny while inhibiting TFs that are incorrectly expressed in differentiated progeny. The latter could be either pluripotency TFs but also TFs for lineages other than the desired lineage that are incorrectly activated during the *in vitro* differentiation process ^43, 72, 77– 81^.

### Chemical engineering of the culture medium

A number of studies have started to test libraries of small molecules to identify factors that enhance differentiation. A good example is pancreatic-beta cell differentiation from PSCs, wherein more than 20 different molecules have been used to create insulin-responsive cells ^82^. Similar examples can be found in the literature for other cell types, including hepatic, neuronal, or cardiac progeny ^36, 83, 84^. However, an often-overlooked characteristic of the culture medium is the nutrient microenvironment. It is, however, well known that medium composition can greatly affect cellular behavior *in vitro*. Several studies demonstrated that nutrient metabolism is one of the major regulators of stem cell fate. For instance, central carbon metabolism plays an important role in PSC maintenance, proliferation, differentiation, and lineage specialization (reviewed in 85). Indeed, PSCs have a glycolytic phenotype ^86^, and failure to induce glycolysis prevents iPSC generation ^87^. By contrast, many differentiated progeny—especially, neural cells ^88^, cardiomyocytes ^89^, and hepatocytes ^90^—are dependent on oxidative phosphorylation for energy production. As the latter is fueled by fatty acids or amino acids or both, it will be paramount to analyze the nutrient requirements of precursor populations generated from PSCs and ultimately develop culture media that support these nutrient needs ^91^ to allow generation of mature lineage-specific cells and simultaneously avoid alternate cell faiths. An example of the latter approach is the eradication of hESCs/iPSCs from cultures by the transient depletion of methionine ^92^.

### Recreation of the stem cell, precursor, and mature cell niche

As discussed above, the ‘purist’ approach to lineage differentiation from PSCs has been to develop differentiation protocols based on monolayer systems coated with a generic ECM layer supplemented with growth factors, morphogens, and cytokines. However, culture in 2D of numerous mature primary cells—such as hepatocytes ^93^ or glia ^46^, to name a few—is incompatible with maintaining a mature, differentiated phenotype *in vitro*. Therefore, it is not surprising that such culture conditions are not suitable for the generation of mature PSC-derived progeny. Hence, researchers are investigating approaches such as co-culture of one or more cell types in 3D culture systems or PSC-derived organoid cultures to better mimic the environment, also named ‘niche’, wherein cell fating and differentiation occur during development and wherein mature cells reside within an organ.

Although organoids from undifferentiated PSCs allow evaluation of the initial steps of cell fating and the spontaneously developing interactions between neighboring cells and their niches, as is thought to occur *in vivo*, it remains difficult to control these steps, and the creation of mature functional cells from stem cell organoids is not easily achieved. A very good example is the self-organizing brain organoid, initially described by Lancaster and Knoblich, that allows the generation of a variety of brain regional identities, including hindbrain, midbrain, forebrain, and even retinal tissue ^94^. However, even if this allows the creation of some of the complexity of the brain, inherent to spontaneous development is that the organoids are highly variable within a single experiment and between experiments, and cells do not achieve a terminally differentiated state.

As an alternative, organoids can be generated from different PSC-derived lineage precursors embedded or not in functionalized hydrogels, in combination with chemical signals (growth factors) to recreate the physicochemical properties of the organ wherein the maturing cells reside. This enables greater control of the cell types present in the 3D culture compared with the spontaneous, less controlled differentiation occurring in undifferentiated PSC organoid cultures ^95, 96^. Until recently, 3D organoid cultures were exclusively performed in matrigel, a poorly defined and largely proteinaceous mixture whose properties cannot be readily modulated and therefore do not allow the evaluation of cell–ECM interactions or do not allow one to understand the role of mechanical forces in cell fate decisions. While many cell types are not intrinsically mechanically active, they encounter and respond to a range of mechanical forces such as shear stress, matrix topography, and rigidity. While we intuitively accept that differentiation induces changes in cell shape, controlling cell shape also affects differentiation ^97, 98^. Mechano-sensation affects cell morphology, cytoskeletal structure, adhesion, and function, emphasizing the importance of cell–ECM interactions ^99, 100^. The advent of highly tunable hydrogels based on poly(ethylene glycol) (PEG) ^101^ or natural gellan gum (GG) ^102^ has significantly increased the flexibility of probing the role of the microenvironment in specifying stem cell fate. A number of teams have developed ECM component libraries wherein matrix stiffness, matrix degradability, and soluble and ECM peptide factors can be combinatorially and systematically tested in high-throughput approaches. This has resulted in the definition of improved PSC culture and differentiation conditions ^103, 104^.

The above-mentioned hydrogel technology not only can be used to embed spontaneous assembly of different cell types but also can be combined with bioprinting, allowing the creation of stem cell niches. Bioprinting, such as bioextrusion ^105^, inkjet bioprinting ^106^, and microvalve-based bioprinting ^107, 108^, has already been used to create 3D tissue and organs from stem cells. However, the spatial resolution of these approaches is in the 50-μm range, not allowing the precise recreation of cell niches. However, this may be feasible when using laser-guided printing ^109, 110^, wherein laser settings and droplet size allow very high-precision printing to the single-cell level. This theoretically should allow one to precisely recreate the cellular niches and to probe the effect of the microenvironment (cell–ECM and cell–cell contact) on cellular differentiation.

As cells also respond differentially to the organization and ultrastructure of the ECM, topographical cues embedded in ECM can further guide tissue development. For instance, hPSC-derived cardiomyocytes grown on microgrooves, that topographically resemble aligned collagen fibers in the developing heart, aligned to the substrate, which resulted in improved sarcomere length ^111, 112^. Therefore, synthetic nanopatterned substrates with well-defined properties are also assessed for their effect on differentiation ^113, 114^.

### Mechanical and electrical engineering

The controlled addition of growth factors and small molecules in conjunction with tailored hydrogels can mimic the physicochemical properties of the immediate *in vivo* environment. However, developing cells are also subjected to electromechanical forces exerted by the organ in which they develop (reviewed in 115). Therefore, a number of studies have started to test the additional effect of electrical or mechanical stimulation (or both) on cardiac and neural differentiation, as electrical activity is a fundamental property of these cell types ^50, 51, 116^. For instance, stimulation paradigms have been well described for both rat and mouse primary cultures ^117, 118^, suggesting that integrating electrical stimulation to enhance the maturation of hPSC-derived neurons is feasible. However, such an approach has not yet been widely applied to human culture systems. Continuous electrical stimulation also improved cardiac differentiation, significantly enhanced connexin expression and sarcomeric structure, and instructed cardiomyocytes to adapt their beating rate ^51^, a sign of electrophysiological maturity ^119^. As electrical activity is coupled to contraction in cardiomyocytes, the role of cyclic stress in cardiac muscle maturation is also being tested. Although applying mechanical stimulation improved the transcriptional and functional profile of hPSC-derived cardiomyocytes ^120^, less is known about the influence of mechanical stimulation on the maturation of other cell types.

### Engineering perfusion

The final and perhaps most arduous addition to the 3D-derived models is to integrate the vascular network, to allow the delivery of nutrients and oxygen to the microtissue and to allow re-circulation of endogenous factors and hence aid in specifying cells within a tissue (for example, zonation in a liver sinusoid). Vascular networks have, for instance, already been printed, integrated into skin tissue or on a cardiac patch ^109, 121^, or incorporated in self-assembling 3D tissues ^96^. When implanted in rodents, the murine blood vessels of the implant connected with the vascular network, integrating blood vessels into the vascular system of the host ^96^. In addition, microfluidic systems have been generated allowing the continuous manipulation of, for instance, pre-defined oxygen gradients, the delivery/removal of specific factors or functional components to/from the microtissue, and the alteration of mechanical forces exerted on the tissue ^122^. For example, Giobbe *et al*. differentiated hPSCs directly on a chip under perfusion to hepatocytes and cardiomyocytes, which presented with significantly increased functionality as compared with unperfused control cells ^123^. Even though perfusion is of such importance for the differentiation of PSCs and their derivatives, a major challenge remains to connect very small (micrometer-diameter) capillaries ^109, 121^ to microfluidic channels that are commonly 5 to 10 times wider than capillaries.

## Conclusions

**Figure 2.**
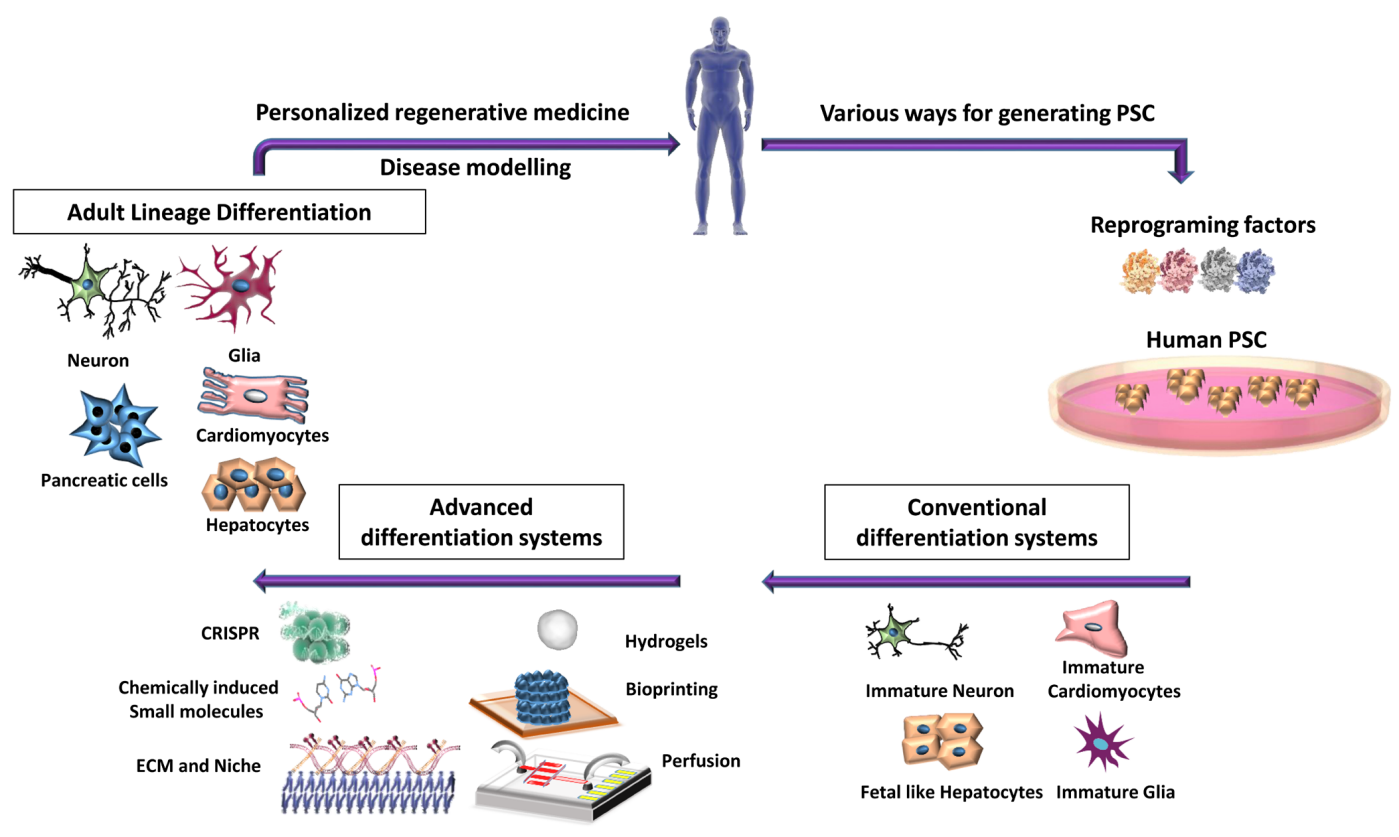
Schematic overview for the advances in lineage differentiation. Induced pluripotent stem cells (PSCs), for studies of human disease or to create differentiated cells for regenerative medicine, can be generated from any somatic cell, from which the desired cell can be differentiated. However, current differentiation systems generate immature progeny. Recent advances in genome editing (CRISPRa/i), chemical screens, and bioengineering—extracellular matrix (ECM) functionalized hydrogels, bioprinting, and microfluidics—are being used to allow the derivation of more mature and functional PSC progeny, which resemble their *in vivo* counterparts better, and can be used for personalized and regenerative medicines. CRISPR, Clustered Regularly Interspaced Short Palindromic Repeats.

The generation of mature PSC-derived progeny, resembling their *in vivo* counterpart, is desperately needed in the field of disease modeling and regenerative medicine. The availability of high-throughput single-cell transcriptomics, computational modeling of gene regulatory networks, and new genome editing tools now allows in-depth characterization of cellular identity and lineage commitment. These advances highlight a previously underestimated heterogeneity in cell populations but in the meantime provide key targets and knowledge of cellular state and function. This increased understanding has been one of the driving forces behind the continuous generation of new differentiation protocols. In addition, progress made in the field of (bio)engineering now allows the creation of hydrogels with mechanical stiffness similar to that of a given organ. It also allows one to decorate the gels with ECM components as well as chemical guides, aside from electromechanical stimuli, to drive differentiation in a 3D configuration. In addition, bioprinting techniques have evolved such that specific ECM topologies that instruct differentiation can be recreated, specific cell-to-cell interactions can be incorporated at the (near) single-cell level, and capillary networks can be co-embedded between different cells to enable continuous nutrient support and removal of breakdown products. All of these tools are starting to recreate different aspects of the niches wherein progenitors and mature cells reside, although much is still to be learned about these niches before we will be able to fully recreate them ( Figure 2).

As we are starting to move away from standard ‘petri dish’ culture systems to high-complexity systems, not only will it be important to ensure reproducibility but also the main challenge will be to adapt these advanced culture systems to medium- and high-throughput formats in a cost-effective way. Thus, even if great strides have been made toward lineage differentiation of stem cells, many challenges are still ahead before it will be possible to generate fully mature and functional cell types.

## Abbreviations

CRISPR, Clustered Regularly Interspaced Short Palindromic Repeats; CRISPRa, Clustered Regularly Interspaced Short Palindromic Repeats-activation; dCas, dead Cas; ECM, extracellular matrix; ESC, embryonic stem cell; hESC, human embryonic stem cell; hiPSC, human induced pluripotent stem cell; hPSC, human pluripotent stem cell; iPSC, induced pluripotent stem cell; PSC, pluripotent stem cell; TF, transcription factor; VP64, virus protein VP16 repeats (4 times repeat); VPR, VP64 fused with p65, a subunit of the ubiquitous NF-κB transcription factor complex (p65) and the Epstein-Barr virus reverse transactivator.
